# In-depth proteomic profiling identifies potentiation of the LPS response by 7-ketocholesterol

**DOI:** 10.1016/j.jmccpl.2025.100285

**Published:** 2025-01-29

**Authors:** Iain R. Phair, Magdalena Sovakova, Noor Alqurashi, Raid B. Nisr, Alison D. McNeilly, Douglas Lamont, Graham Rena

**Affiliations:** a Cellular and Systems Medicine, School of Medicine, University of Dundee, Dundee DD1 9SY, Scotland, United Kingdom; b Centre for Advanced Scientific Technologies, School of Life Sciences, University of Dundee, Dundee DD1 5EH, Scotland, United Kingdom

**Keywords:** Ketocholesterol, Oxysterol, Macrophages, Cholesterol, Inflammation

## Abstract

In patients with stable coronary artery disease, plasma levels of 7-ketocholesterol (7-KC), found at high levels in atherosclerotic lesions, predict risk of incident heart failure dose dependently, potentially contributing to disease aetiology. Previous studies demonstrated that 7-KC can elicit effects on macrophage function; however, effects of 7-KC on the macrophage proteome have not been studied systematically. Here we used quantitative mass spectrometry to establish the effect of 7-KC on the mouse macrophage proteome. 7-KC independently mediated dynamic changes, including on atherogenic/M1 markers, cholesterol metabolism, biosynthesis and transport, as well as nutrient transport more broadly. These changes were however insufficient alone to drive changes in cytokine and chemokine secretion. Rather, they prime the macrophage, potentiating LPS-stimulated TNF alpha secretion and key pro-inflammatory enzymes. Our results indicate that 7-KC has independent metabolic effects on the macrophage; however, effects on the immune system are primarily due to the changes in metabolism priming the response to an inflammatory stimulus. Earlier findings from CANTOS and the recent FDA approval of colchicine highlight that inflammation is a viable target for cardiovascular disease; however, it is currrently unclear which will be the best anti-inflammatory targets to pursue in the future. In this context, our findings suggest that drugs targeting atherogenic markers induced by 7-KC might be well tolerated, as they will not necessarily be expected to be immunosuppressive.

## Highlights

• Effect of 7KC on macrophages has been studied by DIA proteomics.
• 7KC independently regulated atherogenic/M1 markers, cholesterol metabolism, biosynthesis and transport, as well as nutrient transport more broadly.
• 7KC potentiated LPS-stimulated TNF alpha secretion and key pro-inflammatory enzymes.

## Graphical abstract

**Figure:**
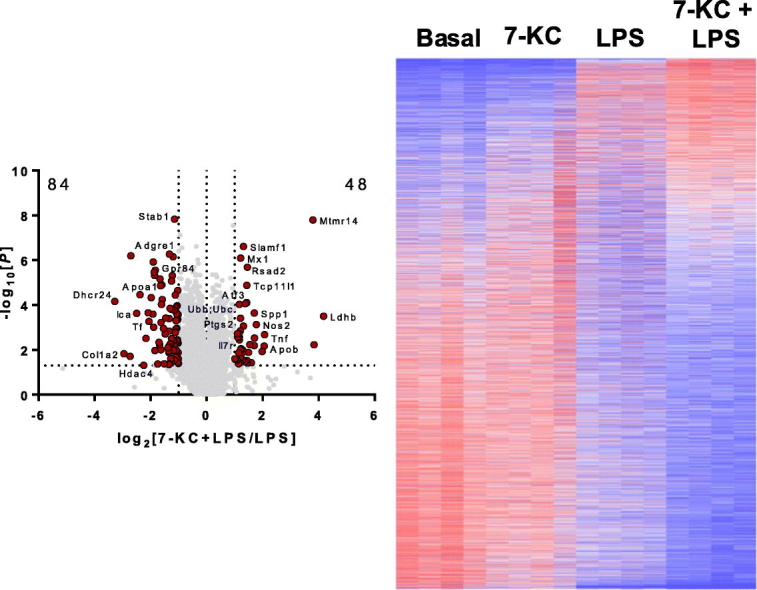

## 1 Introduction

Oxidised cholesterols, or oxysterols, are oxygenated cholesterol derivatives. They are generated through catalytic and non-catalytic processes [1]. Low density lipoprotein (LDL) has a critical physiological role in transporting cholesterol in the blood [2]; however, this becomes dysfunctional in disease states such as insulin resistance. 7-ketocholesterol (7-KC) is the most abundant non-enzymatically generated oxysterol in vivo, as well as being the most chemically stable [3]. Circulating 7-KC levels are elevated in patients with type 2 diabetes compared to healthy volunteers [4] and in patients with atherosclerosis, proportionately to disease severity [5]. 7-KC dose dependently predicts risk of incident heart failure in patients with stable coronary artery disease [5]. 7-KC might thus contribute to disease aetiology. In the human macrophage cell line THP-1, 7-KC promoted cell adhesion and converted macrophages into foam cells [6]. Accumulation of 7-KC in macrophages drives oxidative stress, ER stress and apoptosis [7,8]. Earlier non-systematic studies and a recent systems-level RNA sequencing investigation have found that 7-KC can increase macrophage inflammatory responses [6,[9], [10], [11]]. Targeting of 7-KC action on inflammatory cells is thus seen as a promising intervention target in atherosclerosis and heart failure; however, the overall impact of 7-KC on the macrophage proteome remains unclear. Therefore, in this study, we sought to systematically characterise the impact of 7-KC by state-of-the-art Data Independent Acquisition (DIA) proteomics. We studied bone-marrow derived macrophages due to the importance of TLR4 signalling in progression of atherosclerotic lesions [12]. Our investigation makes the novel insight that the profound metabolic rewiring induced by 7-KC modulates the immune function of these cells, mainly through potentiation of responses to an inflammatory stimulus.

## 2 Materials and methods

### 2.1 Mice

Wild-type C57BL/6 J mice were obtained from Charles River. Animals were maintained under a 12 h:12 h light:dark cycle (holding room lights on at 06:00 and off at 18:00) at 22 ± 1 °C and 50 % humidity. Mice had ad libitum access to standard chow diet (7.5 % fat, 75 % carbohydrate, and 17.5 % protein by energy [RM1 diet; Special Diet Services]) and water. All animal care protocols and procedures were performed in accordance with current regulations. Colonies were maintained under specific pathogen-free conditions, work was approved by local ethical review and carried out subject to a home office licence.

### 2.2 BMDM culture

Primary bone marrow-derived macrophages (BMDMs) were generated as previously described. Briefly, bone marrow was flushed from the femurs and tibia of 6- to 12-week-old C57BL/6 J mice and the bone marrow was passed through a 100 μm cell strainer (Greiner). Macrophages were differentiated for 7 days on bacterial grade plastic in BMDM media: DMEM (Gibco) supplemented with 20 % (v/v) L929-conditioned media as a source of M-CSF, 2 mM Glutamine (Gibco), 10 % (v/v) foetal calf serum (Labtech), 50 μM 2-mercaptoethanol, 10 mM HEPES (Sigma), 100 U/mL Penicillin (Gibco) and 100 μg/mL Streptomycin (Gibco). On day 7, cells were harvested and replated at a density of 500,000 cells/mL on tissue culture-treated plastic in fresh BMDM media. BMDMs were stimulated as indicated in legends with 15 μM 7-ketocholesterol (Cayman Chemical) or 100 ng/mL LPS from *E. coli*, Serotype O55:B5 (Enzo Life Sciences).

### 2.3 Proteomics sample preparation

This was carried out as described previously, see earlier articles [13,14], main methods recapitulated here. All the proteomics data presented is based on the same samples being studied. Peptides were generated using the S-Trap mini column method (Protifi). Briefly, cells were lysed in 4 % SDS, 50 mM TEAB pH 8.5, 10 mM TCEP at room temperature. Samples were transferred to Protein LoBind tubes (Eppendorf), boiled using a ThermoMixer (5 min at 95 °C with 500 rpm on shaker), followed by sonication with a BioRuptor (15 cycles of 30 s on/30 s off). Protein concentration was determined using the EZQ Protein Quantitation Kit (Invitrogen) as per manufacturer's instructions. Samples were alkylated with 20 mM Iodoacetamide (IAA) in the dark at room temperature for 1 h. Lysates were then acidified by the addition of 1.2 % phosphoric acid, then mixed by vortexing. S-Trap binding buffer (90 % HPLC-grade methanol containing 100 mM TEAB pH 7.1) was added to lysates at a 7:1 ratio. Samples were then loaded on S-Trap mini columns, then centrifuged at 4000 *g* for 30 s. Columns were then washed five times with S-Trap binding buffer. Digestion buffer (50 mM ammonium bicarbonate containing trypsin (Promega) at a 1:20 ratio (trypsin:protein)) was added to the top of the column and incubated at 47 °C without shaking for 2 h. Peptides were then eluted by addition and sequential centrifugation at 4000 *g* of 80 μl of digestion buffer, 80 μL of 0.2 % formic acid, and 80 μL of 50 % acetonitrile containing 0.2 % formic acid. Eluted peptides were dried down using a SpeedVac (GeneVac), then reconstituted in 1 % formic acid by incubating on a ThermoMixer at 30 °C for 1 h with shaking at 1000 rpm.

### 2.4 LC-MS analysis

Peptides were analysed on a Q Exactive™ plus mass spectrometer (Thermo Scientific) coupled to a Dionex Ultimate 3000 RS nano (Thermo Scientific). The following LC buffers were used: buffer A (0.1 % formic acid in Milli-Q water (v/v)) and buffer B (80 % acetonitrile and 0.1 % formic acid in Milli-Q water (v/v). An equivalent of 1.5 μg of each sample was loaded at 10 μL/min onto a μPAC trapping C18 column (Pharmafluidics). The trapping column was washed for 6 min at the same flow rate with 0.1 % TFA and then switched in-line with a Pharma Fluidics, 200 cm, μPAC nanoLC C18 column. The column was equilibrated at a flow rate of 300 nL/min for 30 min. The peptides were eluted from the column at a constant flow rate of 300 nL/min with a linear gradient from 1 % buffer B to 3.8 % buffer B in 6 min, from 3.8 % B to 12.5 % buffer B in 22 min, from 12.5 % buffer B to 41.3 % buffer B within 95 min and then from 41.3 % buffer B to 61.3 % buffer B in 23 min. The gradient was finally increased from 61.3 % buffer B to 100 % buffer B in 10 min, and the column was then washed at 100 % buffer B for 10 min. Two blanks were run between each sample to reduce carry-over. The column was kept at a constant temperature of 50 °C. Q-exactive plus was operated in positive ionization mode using an easy spray source. The source voltage was set to 2.2 Kv and the capillary temperature was 275 °C. Data were acquired in Data Independent Acquisition Mode as previously described [15], with some modifications. A scan cycle comprised of a full MS scan (*m*/*z* range from 345 to 1155), resolution was set to 70,000, AGC target 3 × 10^6^, maximum injection time 200 ms. MS survey scans were followed by DIA scans of dynamic window widths with an overlap of 0.5 Th. DIA spectra were recorded at a resolution of 17,500 at 200 *m*/*z* using an automatic gain control target of 3 × 10^6^, a maximum injection time of 55 ms and a first fixed mass of 200 m/z. Normalised collision energy was set to 25 % with a default charge state set at 3. Data for both MS scan and MS/MS DIA scan events were acquired in profile mode.

### 2.5 Mass spectrometry data analysis

Raw mass spectrometry data was processed using Spectronaut (Biognosys) version 15.0.210615.50606 with the DirectDIA option selected. The following parameters were chosen: cleavage rules were set to Trypsin/P, maximum peptide length 52 amino acids, minimum peptide length 7 amino acids, maximum missed cleavages 2. Carbamidomethylation of cysteine was set as a fixed modification while the following variable modifications were selected: oxidation of methionine, deamidation of asparagine and glutamine and acetylation of the protein N-terminus. The FDR threshold for both precursor and protein was set at 1 %. Profiling and imputation were disabled. Quant 2.0 was selected. DirectDIA data were searched against a mouse database from Uniprot release 2020 06. This database consisted of all manually annotated mouse SwissProt entries along with mouse TrEMBL entries with protein level evidence and a manually annotated homologue within the human SwissProt database. Estimates of protein copy number per cell were calculated using the histone ruler method [16].

### 2.6 qPCR

Total RNA was isolated using NucleoSpin RNA Plus mini-isolation kits (Macherey-Nagel) according to the manufacturer's protocols. RNA was reverse transcribed using an iScript cDNA synthesis kit (BioRad) and qPCR carried out as described using SYBR green-based detection methods. Data was normalised to a combination of GAPDH and 18S expression, and primer sequences are shown in Table 1.

**Table 1:** Primer sequences.

Target	Forward	Reverse
Sqle	GTTCGCTGCCTTCTCGGATA	TGATTCAGGTGACTTGGCCC
Hmgcr	TGCGTAAGCGCAGTTCCTT	CACAGTCCTTGGATCCTCCG
18S	GTAACCCGTTGAACCCCATT	CCATCCAATCGGTAGTAGCG
GAPDH	GCCTTCCGTGTTCCTACCC	TGCCTGCTTCACCACCTTC

### 2.7 Analysis of cytokine levels

Following stimulation of cells, the levels of IL-6, IL-10, IL-12p40, MCP-1 and TNF present in the media were determined via a multiplex Luminex-based method (Bioplex, Bio-Rad) using the Bio-Plex 200 system (Bio-Rad).

### 2.8 Statistical analyses

For proteomics data, four biological replicates were generated for each condition. Volcano plot *P* values were calculated using a two-tailed *t*-test assuming unequal variance on log_10_ transformed “copy numbers per cell” values, using Microsoft Excel. A *p* value of <0.05 was considered significant. In single-protein bar graphs comparing two groups only, significance is reported using adjusted *p*-values from the empirical Bayes test in the limma R package, with Benjamini-Hochberg adjustment for multiple testing. A *p* value of <0.05 is considered significant. In bar graphs comparing more than two groups, significance is reported using one-way ANOVA and post-hoc test, also with a p value<0.05 considered significant. To accommodate multiplicity of testing in ANOVA, as we report changes in >30 proteins, we applied a stricter threshold of *p* < 0.01 to the F test. It is worth noting however that in all presented data analysed by ANOVA, the F test was very much lower than that, *p* < 0.0001 for all proteins except PMVK, which was *p* < 0.001. Results in bar graphs are expressed as the mean ± s.d.

Heat maps were generated using the Morpheus tool from the Broad Institute (http://software.broadinstitute.org/morpheus).

For Gene Ontology (GO) Term enrichment analysis, GO terms enriched in proteins with statistically significant changes in levels were identified using the functional annotation tools within DAVID Bioinformatics Resources 6.8, NIAID/NIH (https://david.ncifcrf.gov/). All other analysis is based on at least three separate observations.

## 3 Results

### 3.1 7-ketocholesterol (7-KC) drives dynamic changes in the macrophage proteome

**Fig. 1:**
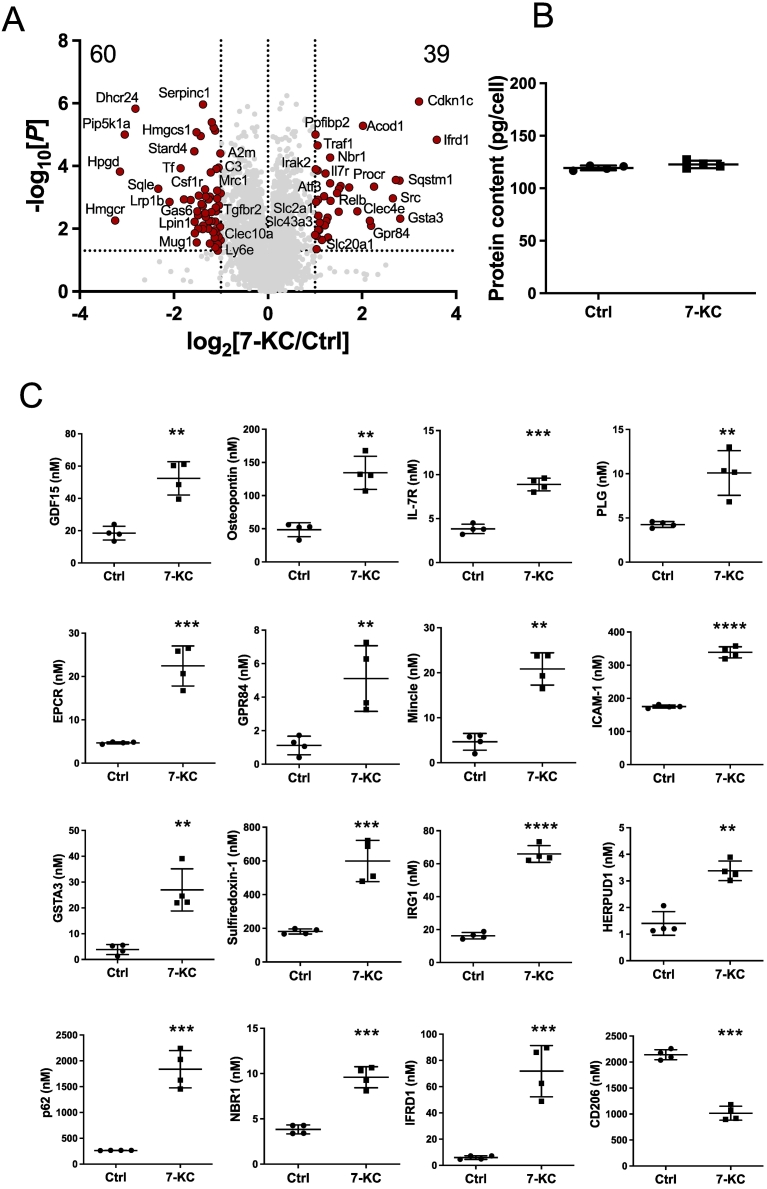

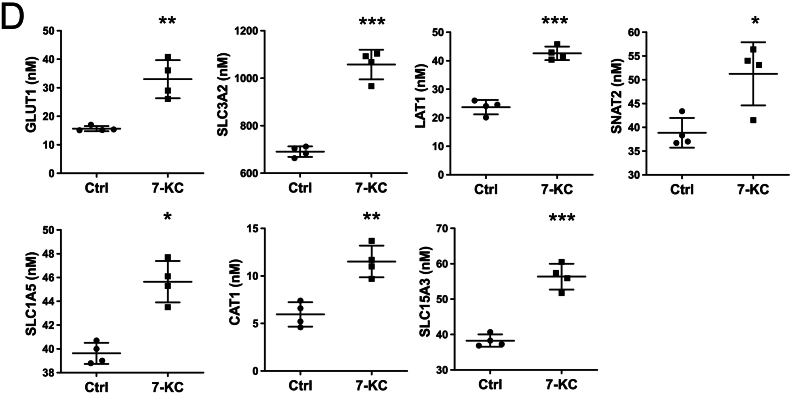
Impact of 7-KC on macrophage proteome. (A) Volcano plot depicts log2 fold change in mean protein copy numbers per cell between untreated BMDMs and those treated with 7-KC for 24 h. Proteins highlighted in red have a significant (*p* < 0.05, *t*-test) fold change <0.5 or > 2. (B) Protein content of BMDMs treated with/without 7-KC for 24 h. (C) Effect of 24 h 7-KC on atherogenic/M1, M2 markers GDF15, osteopontin, IL-7R, PLG, EPCR, GPR84, Mincle, ICAM-1, GSTA3, Sulfiredoxin-1, IRG1, HERPUD1, p62, NBR1, IFRD1 and CD206. (D) Effect of 24 h 7-KC on nutrient transporters GLUT1, SLC3A2, LAT1, SNAT2, SLC1A5, CAT1, and SLC15A3. For proteomics bar graphs statistically significant differences are determined by limma, with *p* values adjusted for multiple testing. **** denotes *p* < 0.0001, *** denotes *p* < 0.001, ** denotes *p* < 0.01, * denotes *p* < 0.05. *n* = 4 dishes per treatment (+/− SD).

To understand the global impact of 7-KC on macrophage function, we carried out quantitative proteomic analysis of bone marrow-derived macrophages (BMDMs) stimulated for 24 h with 15 μM 7-KC. Previous studies indicate that 7-KC concentration in plasma is around 2 μM [17] and in heart failure is increased by 10–30 fold [18], suggesting the concentration we used is relevant to CVD pathology. We identified 6617 proteins and concentrations of each of them were estimated by the proteomic ruler method, using the mass spectrometry signal of histone proteins as an internal standard [16]. 7-KC was observed to induce dynamic changes in the macrophage proteome (Fig. 1a), without effect on protein content (Fig. 1b). Using a fold change cut-off of >2 and *p* < 0.05, we observed increases in the levels of 39 proteins, and decreases in the levels of 60 proteins.

### 3.2 Mechanisms of atherosclerosis

Several of the identified proteins are thought to contribute to atherogenesis, including Growth and Differentiation Factor 15 (GDF15), osteopontin and IL-7R [[19], [20], [21]]; each of which upregulated >2-fold by treatment with 7-KC (Fig. 1c). Plasminogen (PLG) is a zymogen of plasmin, which degrades fibrin clots and haspreviously been shown to associate with atherosclerotic diseases risk in genetic studies [[22], [23], [24], [25]]. More recently it has been shown to have a role in cholesterol efflux from macrophages [26]. EPCR is a suppressor of coagulation [27]. It has been found to be associated with coronary artery disease [28,29]. EPCR is believed to bind lipids and localises to lipid rafts in cell membranes [30]. GPR84 enhances inflammation and phagocytosis in macrophages [31]. Mincle knockout studies suggest that Mincle enhances cholesterol-induced inflammation and foam cell formation [32]. ICAM-1 Knockout studies found that ICAM-1 expression is required for atherosclerotic lesions in ApoE knockout animals with raised cholesterol [33]. Glutathione S-transferase A3 (GSTA3) is a suppressor of lipid hydroperoxide formation [34], whose regulation changes during progression of atherosclerosis [35]. SNPs in GSTA3 are associated with bypass graft failure [36]. Sulfiredoxin 1 (SRXN1) is controlled by NRF2 in bone marrow-derived cells, possibly contributing to pro-atherogenic effects of NRF2 [37]. IRG1 is the enzyme that catalyses production of the anti-inflammatory mediator itaconate [38], which has recently been found to be an important suppressor of atherosclerosis. [39].

Lipophagy is a selective type of autophagy directed towards degradation of lipid droplets rather than proteins, and which is considered a potential treatment target for atherosclerosis [40]. There were three autophagy enzymes regulated by 7-KC. HERPUD1 is a membrane protein situated in the endoplasmic reticulum, which is involved in autophagy and is a negative regulator of cardiac hypertrophy [41]. p62 and NBR1 are both chaperones for autophagy cargo [40,42]. Both are understood to be involved in lipophagy [40,42]. IFRD1 controls osteoclastogenesis in macrophages [43], a process that is known to be affected by obesity [44]. CD206 is a marker of macrophage polarisation to the M2 phenotype. Resolution of recruited inflammatory monocytes to a M2 state is understood to be critical for resolution of atherosclerotic lesions [45]. Remodelling of carbohydrate and other nutrient transport is an important hallmark of insulin resistance, which links obesity with cardiovascular disease. Of note, were increases in the levels of a range of nutrient transporters, including transporters for glucose (GLUT1, SLC2A1), amino acids (4F2(SLC3A2), LAT1, CAT1, SNAT2, CAT1 ASCT2 (SLC1A5)) and dipeptides (SLC15A3) (Fig. 1d).

### 3.3 Cholesterol metabolism and transport

**Fig. 2:**
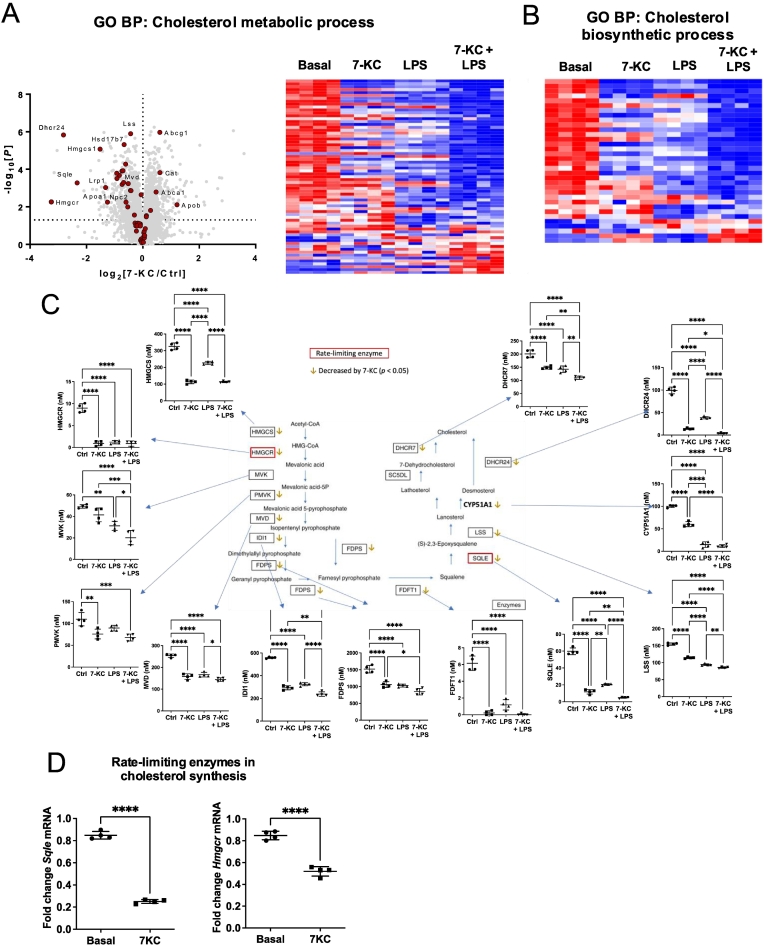
Cholesterol synthesis/metabolising machinery is suppressed in macrophages treated with 7-KC and LPS. (A-B) Volcano plot *(A)* of effect of 24 h 7-KC or LPS, or both agents, on cholesterol metabolising enzymes and heat maps (red is up-regulated, blue is down-regulated) depicting effect of 24 h 7-KC or LPS, or both agents, on cholesterol metabolic *(A)* and cholesterol-synthetic *(B)* processes. (C) Effect of 24 h 7-KC or LPS, or both agents, on individual cholesterol-metabolising enzymes. (D) Effect of 24 h 7-KC on mRNA expression of the two rate-limiting enzymes in cholesterol synthesis. For all proteomics bar graphs, statistically significant differences are determined by one-way ANOVA, with the F test threshold adjusted to 0.01 to account for multiple testing. In 2A and D, statistical significance was determined by *t*-tests. n = 4 dishes per treatment (+/− SD). **** denotes p < 0.0001, *** denotes p < 0.001, ** denotes p < 0.01, * denotes p < 0.05. (For interpretation of the references to colour in this figure legend, the reader is referred to the web version of this article.)

In GO term enrichment analysis, large numbers of proteins decreased by 7-KC treatment in macrophages were associated with various aspects of cholesterol metabolism, including cholesterol biosynthesis and cholesterol influx (Fig. 2a,b). Most enzymes in the cholesterol biosynthetic pathway were decreased, including the rate-limiting enzymes HMG-CoA reductase (HMGCR) and squalene monooxygenase (SQLE), which were also decreased at the level of mRNA (Fig. 2c, d). LPS co-incubation, compared with 7-KC alone, led to potentiation of some cholesterol metabolic enzymes (Fig. 2a-c).

**Fig. 3:**
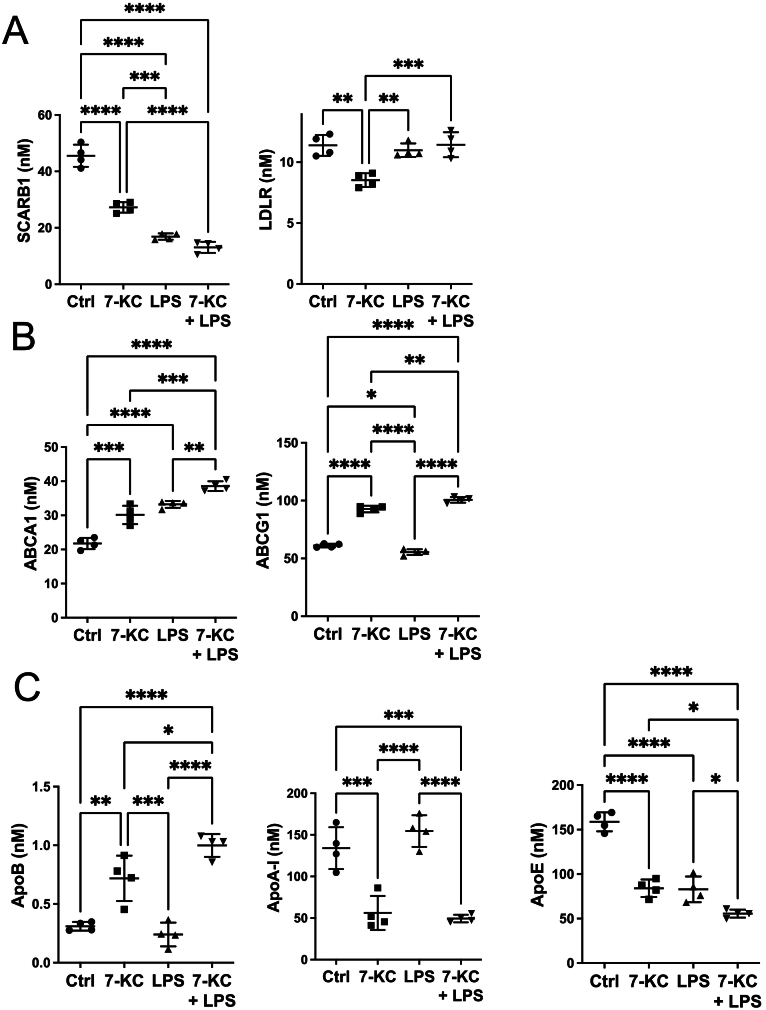
Cholesterol transporters and apolipoproteins are suppressed in macrophages treated with 7-KC and LPS. (A, B) Effect of 24 h 7-KC or LPS, or both agents, on cholesterol influx *(A)* and efflux *(B)* transporters and *(C)* apolipoproteins, are presented. Statistical testing as in Fig. 2. n = 4 dishes per treatment (+/− SD).

Besides changes in cholesterol metabolism, levels of proteins associated with cholesterol influx (SCARB1, LDLR) were decreased by treatment with 7-KC (Fig. 3a). Conversely, transporters known to regulate cholesterol efflux (ABCA1, ABCG1) were elevated by 7-KC treatment (Fig. 3b). ApoB, the primary apolipoprotein of LDL [46], was increased by 7-KC treatment, whilst ApoA-1 and ApoE, components of HDL and other lipoproteins [46], were suppressed by 7-KC treatment (Fig. 3c). LPS also suppressed a variety of cholesterol biosynthetic and metabolic enzymes. There was considerable overlap between proteins targeted by 7-KC and LPS in these GO lists; however a much greater number were changed when the two agents were added in combination (22 proteins changed greater than two fold by combination treatment, compared with 12 for each individual treatment). At individual protein level, LPS and 7-KC co-incubation, compared with 7-KC alone, often led to potentiation of effects (Fig. 2c and Fig. 3b,c).

### 3.4 7-ketocholesterol potentiates LPS-stimulated inflammatory response and cytokine production in macrophages

**Fig. 4:**
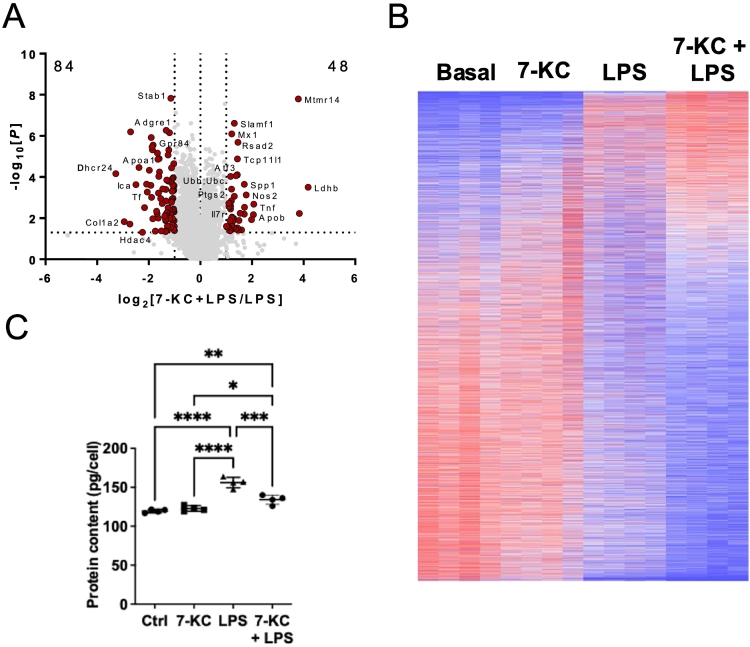
7-KC exacerbates LPS-stimulated inflammatory responses in macrophages. (A, B) Volcano plot *(A)* and global heat map (red is up-regulated, blue is down-regulated) *(B)* of impact of 24 h 7-KC on the response to LPS (C) Effect of 24 h 7-KC on the effect of LPS on BMDM protein content. Statistical testing for (A) as in Fig. 2. and by one-way ANOVA for (C). n = 4 dishes per treatment (+/− SD). (For interpretation of the references to colour in this figure legend, the reader is referred to the web version of this article.)

We then identified proteins where co-incubation with 7-KC elicited large changes compared with LPS alone. Co-incubation of LPS with 7-KC led to >2-fold increase in levels of 48 proteins compared to LPS alone, whilst 84 proteins were decreased in levels >2-fold (Fig. 4 a,b), suggestive that 7-KC elicits dynamic changes on the proteome of LPS-stimulated macrophages. Treatment of macrophages with LPS increased cellular protein content by ~25 % (Fig. 4c); however this was partly ablated by co-treatment with 7-KC.

**Fig. 5:**
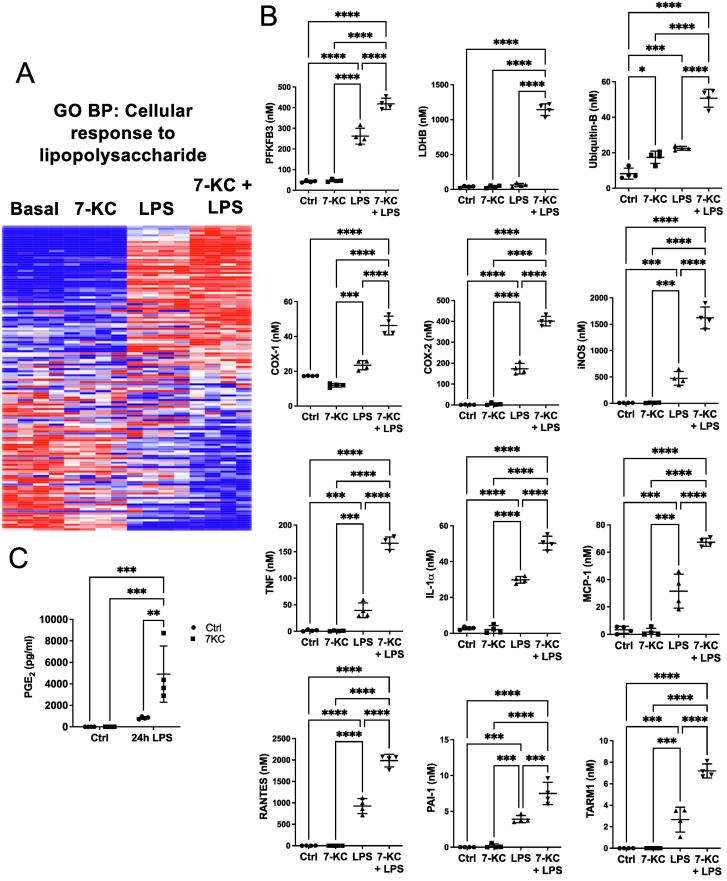
7-KC exacerbates LPS-stimulated inflammatory responses in macrophages. (A, B) Heat map (red is up-regulated, blue is down-regulated) of impact of 24 h 7-KC, or LPS, or both agents, on the GO term gene set ‘Cellular Response to Lipopolysaccharide,’ PFKBP3, LDHB, Ubiquitin B, COX-1, COX-2, iNOS, TNF, IL-1α, MCP-1, RANTES, PAI-1, and TARM1. (C) Effect of LPS and 7KC on PGE_2_. Statistical testing as in Fig. 2. n = 4 dishes per treatment (+/− SD). (For interpretation of the references to colour in this figure legend, the reader is referred to the web version of this article.)

Pro-inflammatory M1 markers and cytokines were strongly represented amongst upregulated proteins (Fig. 5a). LPS upregulates glycolysis in macrophages, which is brought about by induction of the key glycolytic activator 6-phosphofructo-2-kinase/fructose-2,6-bisphosphatase 3 (PFKFB3) [47] (Fig. 5b). Lactate dehydrogenase B, which catalyses oxidation of lactate back to pyruvate, was also upregulated. PFKFB3 was upregulated about two-fold, LDHB exhibited a much stronger synergistic effect.

Pro-inflammatory enzymes were also upregulated by co-incubation of LPS with 7-KC. Prostaglandin E2 (PGE_2_) is produced from arachidonic acid by cycloxygenase (COX) 1 and 2 enzymes in macrophages, in response to inflammatory stimuli [48]. We found the levels of both COX enzymes were induced about two-fold, and secretion of PGE_2_ was even more strongly potentiated by 7-KC (Fig. 5 b,c). LPS also upregulates inducible nitric oxide synthase (iNOS) in M1 macrophages [49]. iNOS is the rate-determining enzyme in nitric oxide (NO) synthesis, which is in turn metabolised to reactive nitrogen species [50] (Fig. 5b). 7-KC potentiated the effect of LPS on inducible nitric oxide synthase by about three-fold (Fig. 5b). 7-KC also potentiated effect of pro-inflammatory cytokines/chemokines TNF, IL-1α, MCP-1, RANTES and PAI-1 (Fig. 5b). Elevated levels of PAI-1 are known to be found in cardiovascular disease [51] (Fig. 5b). The T cell receptor TARM1 was also upregulated (Fig. 5b).

**Fig. 6:**
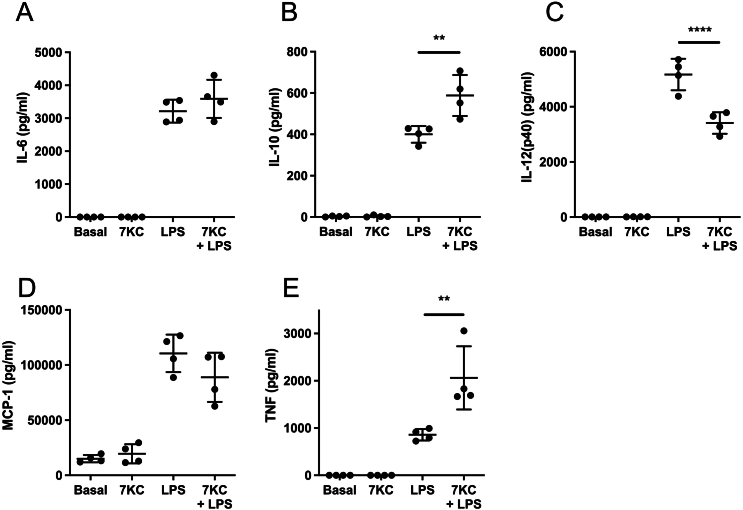
Effects of 7-KC and LPS on cytokine and chemokine secretion. (A-E) Effect of 24 h 7-KC or LPS, or both agents, on secretion of IL-6, IL-10, IL-12p40, MCP-1 and TNF. Statistical testing as in Fig. 2 shown for comparison between LPS alone and LPS + KC. n = 4 dishes per treatment (+/− SD). In each case LPS-induction was significant with respect to basal (p < 0.0001, except TNF, p < 0.05).

We next turned our attention to the impact of 7-KC on pro-inflammatory cytokine production in macrophages (Fig. 6a-e). 7-KC alone was insufficient to induce cytokine production. In contrast, LPS stimulated production of cytokines was modulated by 7-KC. TNF secretion by LPS was particularly enhanced by co-stimulation with 7-KC (Fig. 6e), as was IL-10 (Fig. 6b) and IL-12 was partly ablated (Fig. 6c), whereas in contrast two other cytokines we measured, MCP-1 and IL-6, were unaffected by co-incubation with 7-KC (Fig. 6d,e).

### 3.5 Antioxidant enzymes

Previous studies have demonstrated that 7KC induces ROS production [52]. Besides upregulation of GSTA3, SRXN1 described earlier, 7KC triggered a broader remodelling of redox enzymes involved in detoxification of ROS (Supplementary Fig. 1). We observed the upregulation of a range of antioxidant enzymes, including catalase, glutathione modifying enzymes (GSR, GSTT1, MGST1) haptoglobin (HP), peroxiredoxin-1, 2 and 5 (PRDX1,2,5), superoxide dismutase 2 (SOD2) and Thioredoxin reductase-1 (TXNRD1), whilst other enzymes were downregulated.

## 4 Discussion

### 4.1 7-ketocholesterol drives dynamic changes in macrophage metabolic enzymes and atherosclerosis markers

In the current study, we identified independent effects of the oxysterol 7-KC on metabolic enzymes and atherosclerosis markers. Previous studies in other cell types have shown that treatment with 7-KC suppresses cholesterol synthesis [53]. Our systems-level results were consistent with the previous work, as we observed suppression of the two rate-determining enzymes in the pathway at protein and mRNA level. There was considerable overlap between individual cholesterol metabolic proteins targeted and in addition, an additive effect when the two agents were added together. 7-KC also increased the levels of cholesterol efflux transporters ABCA1 and ABCG1. ABCA1 efflux is PLG-dependent [54], which we also observed was upregulated. Previous studies have also found efflux of 7-KC is mediated at least in part by ABCG1 [55], suggesting that the upregulation that we saw following 7-KC treatment is a feedback mechanism enabling 7-KC-loaded cells to remove intracellular 7-KC. The additional effects of 7-KC that we observed on amino acid and glucose transporters are likely to be aimed at mitigating effects of fat overloading, for example, suppression of mitochondrial oxidation of other substrates [56]. In addition to these metabolic effects, 7-KC had independent effects on markers of M1 polarisation, many of which are also considered to be atherogenic markers, including GPR84, Mincle, ICAM-1, IRG1 and p62. The synergistic effect of LPS and 7-KC on PFKFB3 further suggests that 7-KC magnifies the glycolytic switch triggered by LPS. We previously showed that induction of all six of these proteins by LPS is exaggerated in AMPK knockout M1 macrophages [14]. Taken together, these findings suggest that metabolic stress induced by 7-KC contributes to the changes in protein levels we observed. 7-KC also down-regulated the M2 marker CD206, consistent with 7-KC favouring a pro-inflammatory macrophage phenotype.

7-KC also regulated p62 and NBR1, which control lipophagy, considered a potential treatment target for atherosclerosis [40,42]. The substantial effect of LPS and 7-KC on LDHB, which was unaffected by either agent alone, is currently unexplained; however, both LPS and 7-KC treatment may both be considered metabolic stressors, as well as generators of ROS. Perhaps both hits are required to impact LDHB. Since LDHB catalyses lactate oxidation, the induction of this enzyme may enable the cell to maintain pyruvate levels if these become limiting for essential cellular functions.

The effects of 7-KC on ApoB and ApoA-1, which are not thought to be expressed by macrophages, are interesting and will require further investigation. Identification of these apolipoproteins is unlikely to be due to serum contamination and previous studies have established that macrophages scavenge extracellular lipoproteins [57].

### 4.2 7-ketocholesterol is insufficient to affect cytokine secretion, acting instead by modulation of LPS responses

The DIA methodology is sensitive enough to identify low abundance proteins [13], which in this study enabled us to study cytokines. In principle, 7-KC could affect macrophage activity and cytokine secretion independently, as in some of the effects on atherogenic markers and metabolic pathways described above. Alternatively, it could act on cytokines by modulating inflammatory stimuli. Our investigation strongly supports the second scenario. The action of 7-KC on macrophage function and cytokine secretion is indirect, as it depends on a potentiation of inflammatory mediators such as LPS. By itself, 7-KC was almost entirely without effect on cytokines and pro-inflammatory enzymes, whereas in contrast, there was a striking potentiation of many effects of LPS. This included potentiation of COX levels and PGE_2_ production, as well as iNOS levels.

Investigating the ability of 7-KC to potentiate the inflammatory effect of LPS, 7-KC potentiated TNF protein levels and secretion, as well as other cytokines regulated by TNF, including IL-10, which was also increased. Previous studies in human macrophages found that IL-10 is increased after chronic stimulation with TNF [58]. IL-10 is typically thought of as an anti-inflammatory [59], or resolving cytokine, overall in our dataset the pro-inflammatory effects of 7-KC were dominant. Other classical inflammatory stimuli besides TNF can affect both ‘pro-inflammatory’ and ‘anti-inflammatory’ cytokines [60], and the 7-KC potentiation also affected both, suggesting that metabolic changes may be key to each of them. IL12p40 was reduced with 7-KC. This finding is consistent with earlier findings that TNF can suppress IL12p40 production when mice are injected with *Corynebacterium parvum* [61]. In contrast other cytokines we studied, including IL-6 and MCP-1, were far less affected by co-incubation of LPS with 7-KC. Further work will be required to understand the mechanisms involved in the effects of 7-KC that we have studied; however, we observed a number of changes in enzymes regulating ROS, that may contribute.

In terms of limitations, we feel that BMDMs are a very good model for these studies due to the importance of TLR4 signalling in progression of atherosclerotic lesions [12]; however, further work will be required to validate key findings in lesional macrophages. It will also be interesting to harness the DIA approach to study different cell types, as for example, previous studies have found effects of 7-KC on human umbilical vein endothelial cell stress [62]. We chose only one time point of 24 h because of our interest in long-term effects and previous observations that the LPS response is sustained in the 24-72 h time frame [63]; however, a single short-term time point (24 h) will not necessarily account for all long-term effects. Examining multiple time points could provide further insights into dynamic changes over time. In addition, it will be important to address impact of 7-KC on other immune cell types.

### 4.3 Targeting inflammation for cardiovascular disease

The CANTOS trial of canakinumab established that targeting inflammation can improve cardiovascular outcomes [64]. Recently, colchicine became the first Food and Drug Administration approved medication for atherosclerotic CVD [65]. Both these drugs act on IL-1β [66] and other drugs affecting this target have also been investigated for repurposing, including work from our group [67,68]. Inflammation is highly complex however and this complexity lends itself to systems-level analysis. Despite this, there is a paucity of systems-level investigation that might give a more granular insight into the best additional inflammatory targets to pursue beyond IL-1β, a gap that our current research is addressing. Our study adds to earlier evidence that control of IL-1β and TNF becomes dysregulated in response to oxidised lipids [69,70] and evidence linking changes in IL-10 and IL-12 to atherosclerosis development [71,72]. Together, these findings support further research into regulatory mechanisms underlying potentiation of expression of these cytokines, towards identifying additional CVD drug targets.

## 5 Conclusion

Our results demonstrate that 7-KC independently mediated sometimes profound changes in macrophage metabolic enzymes, as well as atherogenic/M1 markers; however, 7-KC did not have an independent effect on COX enzymes, PGE_2_, iNOS and cytokine/chemokine secretion. Instead, 7-KC acts on these latter aspects by modulating the response to LPS. In terms of drug treatment, this suggests that drugs targeting atherogenic markers induced by 7-KC might be well tolerated, as they will not necessarily be expected to be immunosuppressive. Our work adds to the increasing body of evidence, including CANTOS and the recent FDA approval of colchicine for CVD, that pharmacological targeting of the immune system is a druggable target for CVD.

The following is the supplementary data related to this article.

Supplementary Fig. 1 Heat map (red is up-regulated, blue is down-regulated) of impact of 24 h 7-KC or LPS, or both agents, on enzymes involved in detoxification of reactive oxygen species. *n* = 4 dishes per treatment (+/− SD). Supplementary Fig. 1

## CRediT authorship contribution statement

**Iain R. Phair:** Writing – review & editing, Writing – original draft, Methodology, Investigation, Formal analysis, Conceptualization, Supervision. **Magdalena Sovakova:** Writing – review & editing, Investigation. **Noor Alqurashi:** Investigation, Writing – review & editing. **Raid B. Nisr:** Data curation, Writing – review & editing. **Alison D. McNeilly:** Writing – review & editing, Investigation. **Douglas Lamont:** Writing – review & editing, Investigation, Data curation, Conceptualization. **Graham Rena:** Writing – review & editing, Writing – original draft, Supervision, Project administration, Funding acquisition, Formal analysis, Conceptualization.

## Funding

We thank 10.13039/501100000274BHF PG/18/79/34106, 10.13039/501100000361Diabetes UK 19/0006045, 10.13039/501100000265MRC and the 10.13039/501100011820Saudi Arabian Cultural Bureau for supporting this research.

## Declaration of competing interest

The authors declare the following financial interests/personal relationships which may be considered as potential competing interests: Graham Rena reports financial support was provided mainly by 10.13039/501100000274British Heart Foundation, the rest provided by the other funders shown. If there are other authors, they declare that they have no known competing financial interests or personal relationships that could have appeared to influence the work reported in this paper.
