# Supplementary figures and images for: In-depth proteomic profiling identifies potentiation of the LPS response by 7-ketocholesterol
Source: J Mol Cell Cardiol Plus. 2025 Jan 29;11:100285. doi: 10.1016/j.jmccpl.2025.100285 (PMC11847031; doi:10.1016/j.jmccpl.2025.100285)

Supplementary Figure

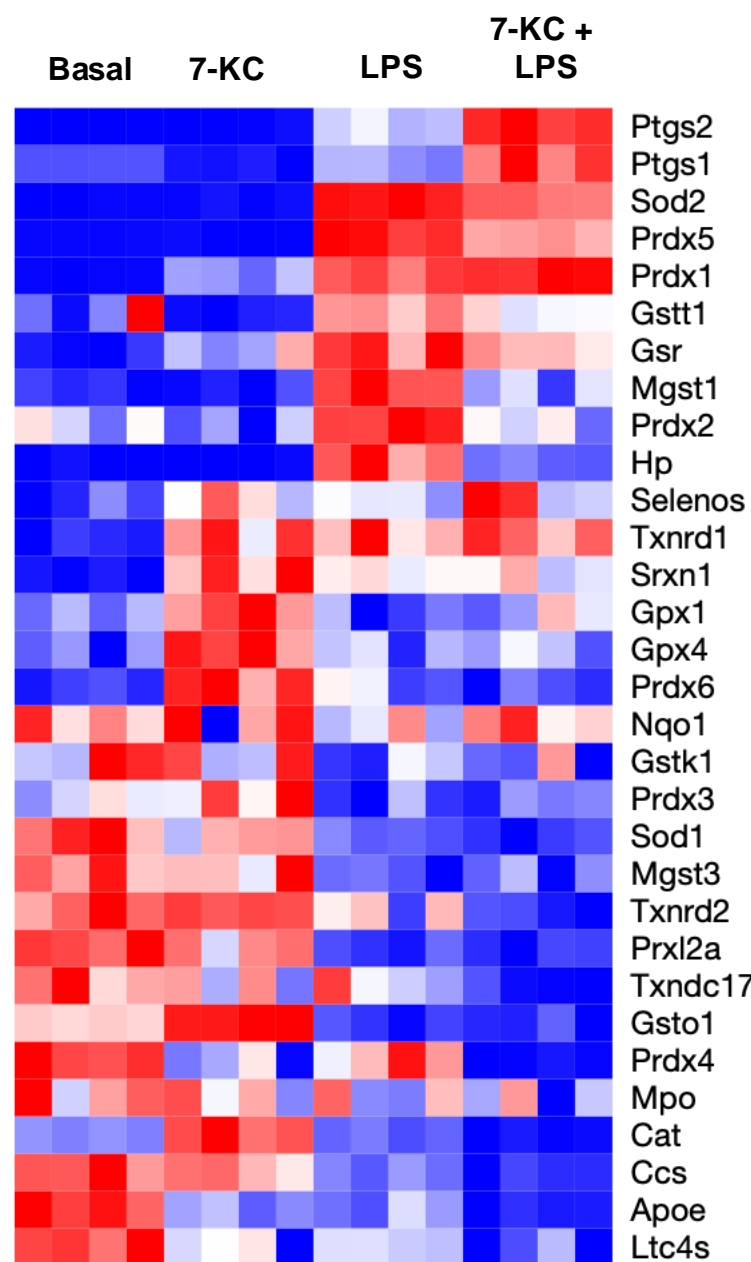

Supplement: Supplementary Fig. 1 — Heat map (red is up-regulated, blue is down-regulated) of impact of 24 h 7-KC or LPS, or both agents, on enzymes involved in detoxification of reactive oxygen species. n = 4 dishes per treatment (+/− SD). [file mmc1.pdf]
